# Clinical Features of Acquired Esotropia and Surgical Management in a Real-World Cohort of Myopic Patients

**DOI:** 10.1097/WNO.0000000000002520

**Published:** 2026-09-18

**Authors:** Michael J. Gilhooley, Omran Sarwani, Gillian Johnson, Indran Davagnanam, Aditi Das, Katie Williams

**Affiliations:** Department of Neuro-ophthalmology (MJG), Jules Stein Eye Institute, University of California, Los Angeles, Los Angeles, California; The Institute of Ophthalmology (MJG, AD, KW), University College London, London, United Kingdom; Departments of Strabismus (MJG, AD, KW), Radiology (OS) and Translational Neurosciences & Stroke. Moorfields Eye Hospital, London, United Kingdom; Department of Radiology (OS, ID), National Hospital for Neurology & Neurosurgery, Queen Square, London, United Kingdom; School of Allied Health Professions and Nursing (GJ), Institute of Population Health, University of Liverpool, Thompson Yates Building, The Quadrangle, Brownlow Hill, Liverpool, United Kingdom; Great Ormond Street Hospital for Children (KW), Great Ormond Street, London, United Kingdom; and Section of Ophthalmology (KW), School of Life Course & Population Sciences, King's College London, Strand, London, United Kingdom.

## Abstract

**Background:**

The global increase in myopia prevalence has led to greater interest in associated esotropias. While myopic patients can present with common or special forms of strabismus (e.g., “heavy eye syndrome”), the relative incidence of associated strabismus and best surgical approaches are not widely understood. The purpose of this study was to present a “real-world” cohort of myopic patients with esotropia to determine what diagnostic features are important and how best to treat esotropia in myopia.

**Methods:**

A retrospective case-note review of adults seen at Moorfields Eye Hospital identified 68 cases as follows: age ≥ 18 years, spherical equivalent ≥3.00D, and diagnosis of esotropia. Demographics, orthoptics, refraction, MRI axial length and angle between the lateral and superior rectus muscles (LR:SR angle), surgical technique, and 3-month postoperative outcomes were recorded.

**Results:**

At presentation, mean age = 42.4 ± 2.2 years, median refraction = −8.25D, median deviation = 20 prism dioptres (PD) (near and distance) horizontal and vertical 4PD near and 3.5PD distance. There was no correlation between horizontal deviation and spherical equivalent (r = −0.044, *P* = 0.76). Median axial length was 27.30 mm and LR:SR angle 107.5° by MRI. Thirty-seven patients underwent surgery, 29 unilateral horizontal recess-resect (n = 9/29 fixed sutures, n = 20/29 adjustable), and n = 1 Yokoyama. Median postoperative horizontal deviation was 6PD near (all esodeviation) and 3PD distance (20% exodeviation, 80% esodeviation, 2 patients had a postoperative vertical deviation, and n = 9 required a second procedure).

**Conclusions:**

In this large, real-world, clinically and radiographically characterized cohort of myopic patients, the incidence of special strabismus was low and there was no association between refraction and size of deviation. Unilateral recess-resect procedures provided good outcomes with both adjustable and fixed sutures. This cohort will aid in the everyday management of this increasing clinical problem and form the basis of the further randomized, prospective trials that are greatly needed in this area.

## Abstract 2

Supplemental Digital Content is Available in the Text.

Myopia is among the most common ophthalmic conditions worldwide^1^ and has been observed to be steadily increasing in prevalence over recent years internationally.^2–4^ The risk of ocular complications in myopia (e.g., retinal detachment, myopic maculopathy)^5–7^ is exponentially increased with the degree of myopia. With rising prevalence, the burden of these associated diseases is predicted to rise including, of particular relevance to the neuro-ophthalmologist, strabismus and ocular misalignment.^8^

A familiar manifestation of ocular malalignment in myopia is that of acquired distance esotropia, also known as the “*heavy eye syndrome* (HES),”^9^ a specific subset of esotropia related to myopia. This has classically been described as a combination of progressive esotropia and hypotropia with limitation of abduction and elevation. While there are no universally accepted diagnostic criteria in this condition, esotropia with high axial length and displacement of the rectus muscles are commonly used to define the cases and have been proposed as potential diagnostic criteria. Orbital imaging techniques (computed tomography^10^ and MRI ^11^) have allowed theories of pathogenesis to be proposed. These emphasize a superior–lateral displacement of the globe due to laxity of the ligamentous band connection between lateral rectus (LR) and superior rectus (SR) muscles allowing inferior displacement of the LR and superior–medial displacement of the SR.^12^

The related syndrome of age-related distance esotropia (or *sagging eye syndrome* [SES])^13^—originally described in nonmyopic, elderly individuals—has also been seen in cohorts of myopic patients and is likely to have an overlapping pathogenesis.^17^ It would appear, though, that lateral rectus displacement—due to SR-LR band laxity—is less prominent in this condition than in HES with minimal SR displacement and no displacement of the globe. This has been proposed to translate to a more acute angle between the SR and LR (SR:LR angle) on MRI studies in SES than in HES.^17^ This difference in the relative displacement of the globe is significant for surgical planning, with Yokoyama et al^15^ describing a surgical technique to specifically address displacement of the muscles and so both horizontal and vertical deviation.

While special forms of strabismus—such as HES, SES—are seen in myopic patients, other causes are likely to be prominent as well. Therefore, the purpose of this study was to present a large, real-world cohort of patients with moderate and high myopia concurrently diagnosed with esotropia. This will allow important questions to be answered: what features are common in myopes presenting with esotropia, how common are special forms of strabismus, and what are the implications of this for physicians seeing adults with strabismus?

## METHODS

### Identifying Cases

A retrospective case-note review of adults seen in the Moorfields hospital system between 2018 and 2023 was performed as summarized in Figure 1. Patients were identified from the electronic medical record (EMR) (OpenEyes Across Health, Ghent, Belgium). Inclusion criteria included the following: age >18 years, a spectacle prescription of ≥ −3.00D spherical equivalent, and a diagnosis of acquired esotropia (acquired in this context referring to a strabismus presenting for the first time in adulthood [after the age of 18 years], rather than a primary, pediatric strabismus; this definition was kept as broad as possible to identify as many cases as possible and incorporates all modes of progression, duration, and [adult] ages of onset). A total of 68 patients met these criteria and proceeded to further investigation.

**FIG. 1.**
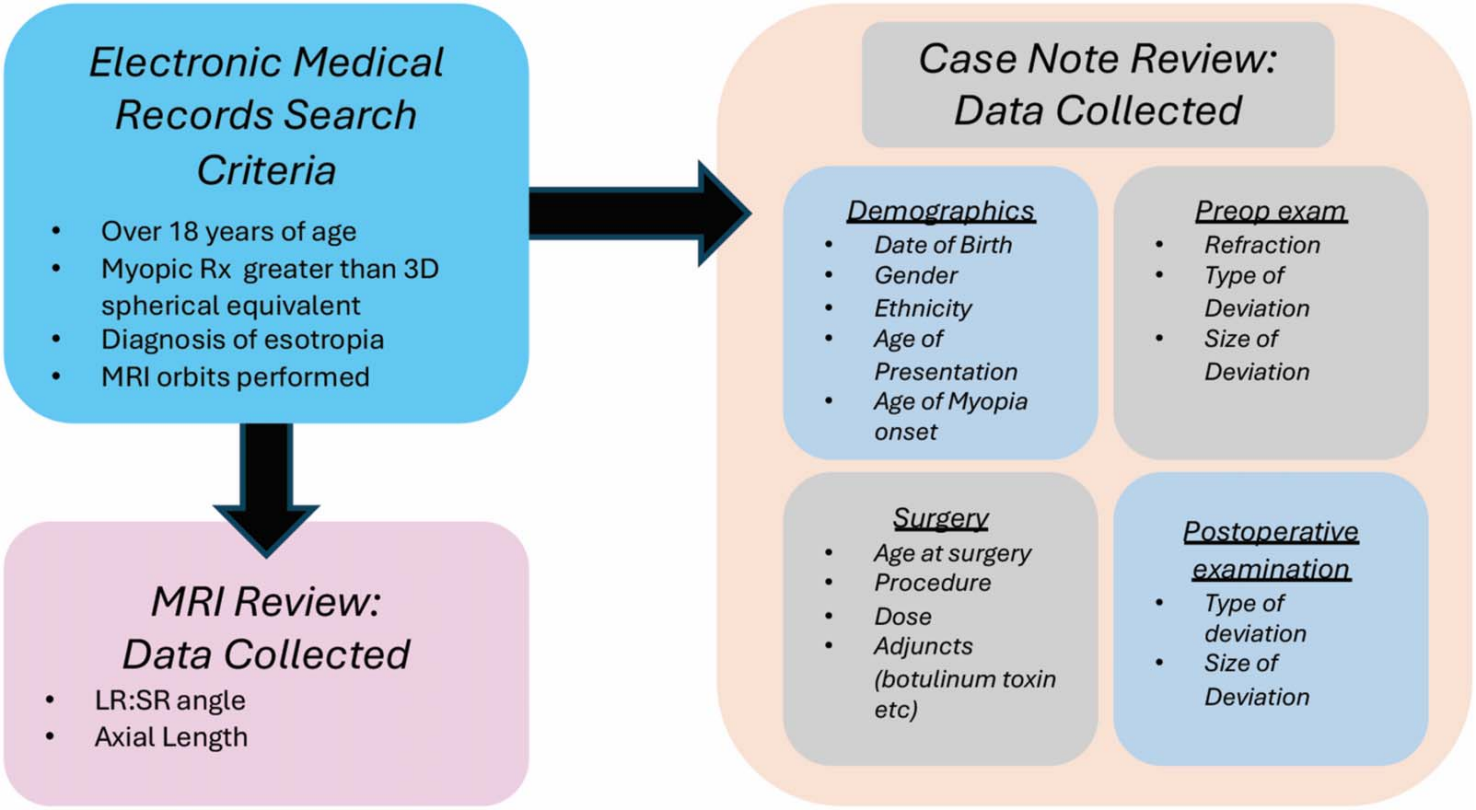
A flowchart describing the EMR search strategy, data collected during the case-note review, and approach to review of the MRI images. EMR indicates electronic medical record; LR:SR angle, angle between the lateral and superior recti muscles.

### Data Collection

Data were collected by qualified researchers (orthoptists or ophthalmologists with subspeciality training in strabismus) by direct consultation of the EMR or review of paper medical records where necessary.

Demographic data including date of birth, age, sex, ethnicity, date of presentation, and age at presentation were collected. In addition, refractive information from the record included the sphere and cylindrical prescriptions from which the spherical equivalent was calculated using the standard formula (sphere + [cylinder/2]) and the best-corrected visual acuity for each eye.

Orthoptic information was recorded including the direction, magnitude, and type (manifest or latent; horizontal or vertical) of deviation before surgical intervention and at the 3-month postoperative visit. Age at surgery and type of operation performed were similarly extracted as was information regarding the use of adjustable sutures, the need for reoperation, or use of nonsurgical interventions (prisms or botulinum toxin) either as primary therapeutic or as adjunctive procedure.

### Radiographic Examination

High-resolution, clinical, T1 or T2 imaging of the orbits was performed using 1.5 and 3T scanners with volumetric sequences obtained depending on the clinical indication of the scans. Images were reconstructed in the quasi-coronal planes (perpendicular to the orbital axis) on dedicated VuePACS radiographic workstations (Phillips, Farnborough, Surrey, United Kingdom). The data provided by Clark et al^17^ (on the normal anterior location of the rectus pulleys), were used as a general reference to locate the pulleys in the coronal plane; however, as our acquired images and sequences were based on various clinical indications which on occasions were not directly related to a globe/orbital pathology, as opposed to being dedicated research based orbital scans, the resolution and certain techniques from this paper were not replicated. This methodology, although based on those used in research settings and standardized to the degree possible with clinical data, was a reflection of what data would actually be available to clinicians in real life in the clinic. Magnetic resonance images of the orbits were reviewed by 2 neuro-radiologists, and an agreement was reached by consensus on the key structural locations where the required measurements should be obtained. Using the data provided by Tan and Demer^17^ who used an automated technique to locate the centroids of the rectus muscles and the globe, the centroids were located manually on a VuePACS workstation and quasi-coronal images were reconstructed 8 mm anterior to the junction of the globe and the optic nerve. Lines were drawn connecting the respective muscle centroids to the globe centroids. The angle formed between the 2 lines: one connecting the centroid of the SR muscle to the globe centroid (GC) and the other connecting GC to the centroid of the LR muscle, was defined as the “superior rectus-lateral (SR-LR) displacement angle.” This manual method was used in place of the automated centroid detection used in the reference study due to the clinical nature and variable resolution of our imaging data. Axial lengths of the globes were obtained using the axial MRI sequences measuring the distance between the anterior corneal and the anterior retinal surfaces. Where images were of too poor quality to perform this analysis, or were not available in the institutional PACS at all to review, patients were excluded from the radiographic analysis.

MRI scans from 5 patients without strabismus were similarly analyzed for illustrative purposes, but not directly compared with the myopic group. A random sample of 10 cases were sent (masked) to a second neuroradiologist with a copy of the analysis protocol, and the values attained by the second examiner were correlated with those of the first on this subsample.

### Statistical Considerations

Prism (V10.4.1, GraphPad, San Diego, CA) and Stata (V18.0, StataCorp, College Station, TX) were used for statistical analysis, linear regression, and the production of graphs. Data are presented as mean ± standard error of the mean where a normal distribution is confirmed and as a median and interquartile range where it is not. The Kolmogorov–Smirnov test was used to determine normality. For non-normal, paired data, the Wilcoxon test was used to compare means; for normally distributed data, a paired *t* test was used. For unpaired, non-normal data, the Mann–Whitney test was used. Linear regression models were used to look for significant associations with multiple covariates. This study adhered to the tenets set out in the Declaration of Helsinki and was deemed exempt from ethical review by the National Health Service Health Research Authority.

(See **Supplemental Digital Content**, http://links.lww.com/WNO/B23).

## RESULTS

### Demographics

The described search strategy found 68 patients: 54% were female and 41% male with a mean age of 42.4 ± 2.2 years at presentation; details of demographics are provided in Figure 2. Visual function was generally very good with a median best-corrected visual acuity (BCVA) in the better eye of 0.00 logarithm of the minimum angle of resolution (Fig. 2D) and no significant difference in BCVA between left and right eyes (Wilcoxon test, *P* = 0.8798). There was a strong correlation between spherical refraction in the right and the left eye (r = 0.81 *P* = <0.01); therefore, data on refraction are presented as the mean of left and right eyes, the median value of which was −8.25D (Fig. 2).

**FIG. 2.**
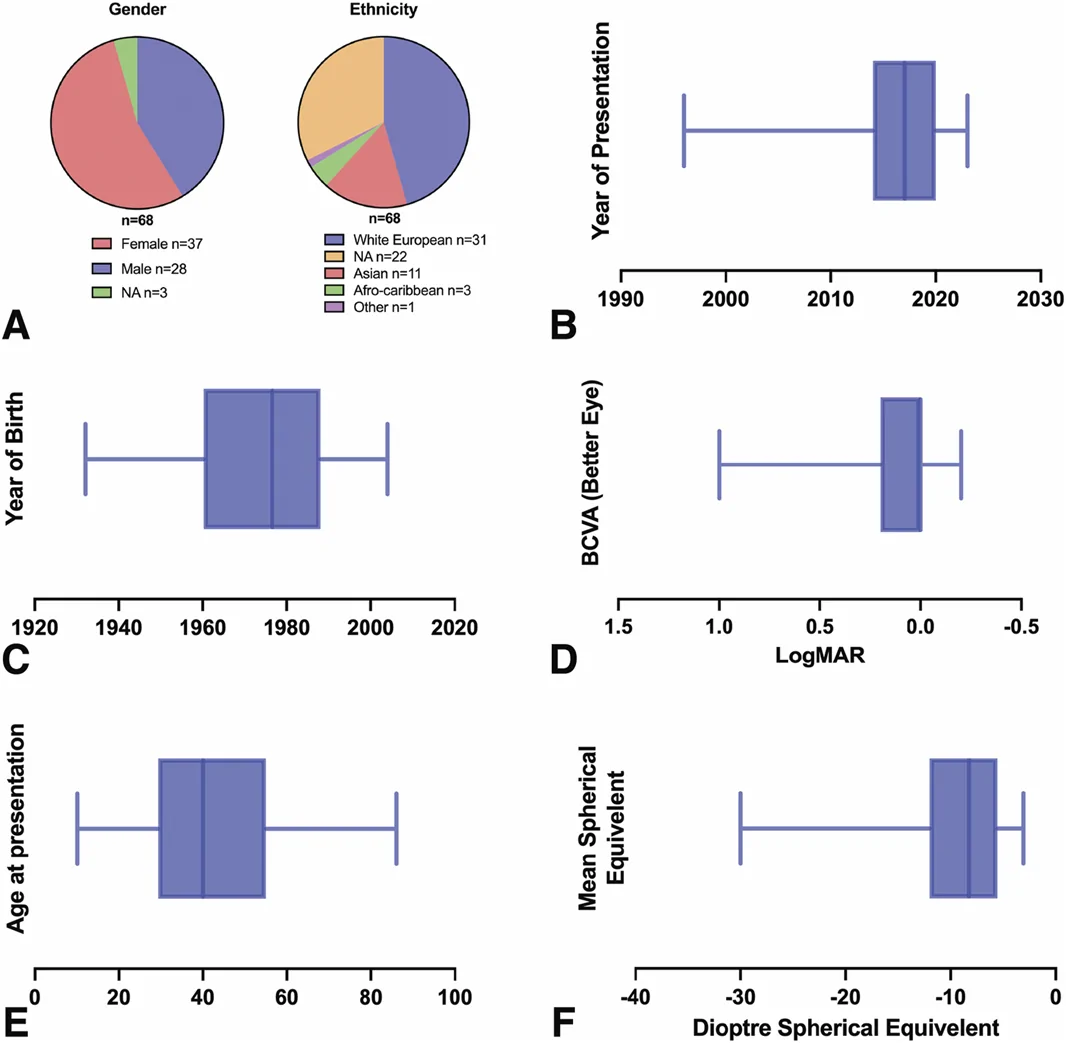
n = 68 cases were found as a result of the EMR search described in Figure 1, and the demographics of the cohort are summarized in this figure. **A**. Gender and ethnicity distribution. **B**. Year of presentation. **C**. Year of birth. **D**. Best-corrected visual acuity of the better seeing eye. **E**. Age at presentation. **F**. Mean spherical equivalent over both eyes. EMR indicates electronic medical record; NA, data not available.

### Orthoptics

Information on the deviation at first visit was extracted from orthoptic reports (Fig. 3). While a majority had a manifest deviation for near (n = 36, 52%, Fig. 3A), a larger proportion were manifest in the distance (n = 54, 79%) and with a significantly larger total magnitude of deviation in the distance than near (Wilcoxon test, *P* = 0.0027). Manifest vertical deviations (Fig. 3B) were similarly less common for near (n = 9, 13%) than for distance (n = 17, 25%). Where they were present, there was no significant difference between vertical deviations at near and in the distance (median: near 4 prism dioptres [PD], distance 3.5PD; Wilcoxon test, *P* = 0.9531). There was no significant association between near horizontal deviation (beta −0.21, *P* = 0.76) or distance horizontal deviation (beta = −0.30, *P* = 0.66) and mean spherical equivalent, adjusted for age and sex (Fig. 3C). Two patients were labeled specifically in their notes as carrying a diagnosis of “heavy eye syndrome.”

**FIG. 3.**
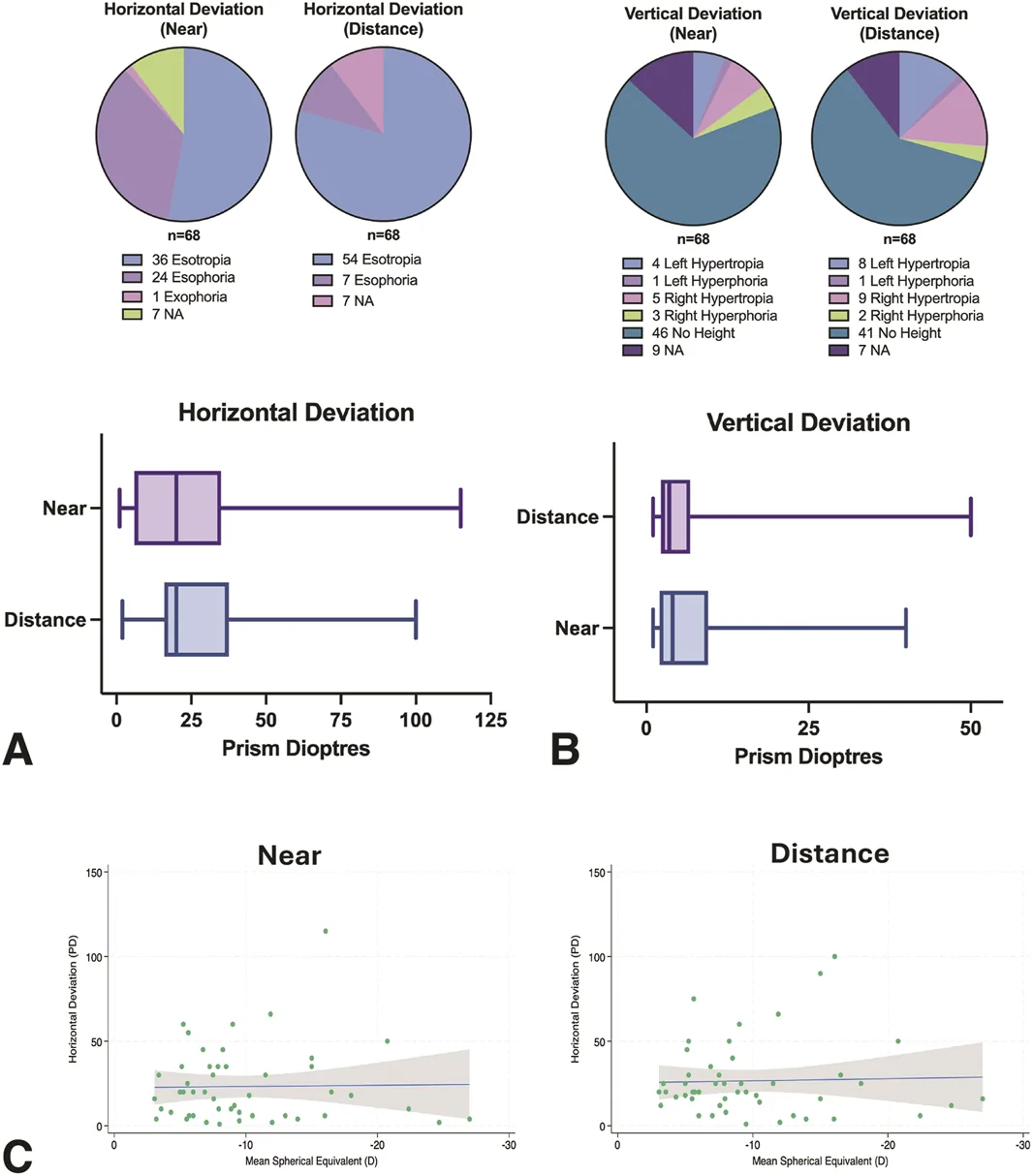
Baseline orthoptic metrics. **A**. Pie charts representing the proportion of patients with manifest and latent deviations for near and distance. Whisker plots describing magnitude of total deviation at near and distance. **B**. Similarly for vertical, whisker plot shows only nonzero values (the majority of cases had no height). **C**. Linear regression was performed, controlled for age and sex without an association being demonstrated between spherical equivalent refraction and the size of horizontal deviation when adjusted for age and sex. NA indicates data not available.

### MRI

In our cohort, n = 32 had available MRI imaging of the orbits (Fig. 4) of sufficient quality to make appropriate measurements. In these cases, there was strong correlation between superior:lateral rectus angles in right and left eyes (r = 0.73, *P* = <0.01); therefore, we present the mean of both eyes within each subject, giving a median of 107.5° across subjects. Similarly, there was good bilateral correlation in radiographic axial length (r = 0.92, *P* = <0.01) with a median value of 27.3 mm. The nonstrabismus scans returned a mean SR:LR angle of 96.22 ± 1.23° and axial length of 23.94 ± 0.35 mm. There was an excellent interobserver correlation for all variables: SR:LR angle right (r = 0.92, *P* = <0.001), left (r = 0.99, *P* = <0.0001) and axial length right (r = 0.98, *P* = <0.0001), left (r = 0.98, *P* = <0.0001).

**FIG. 4.**
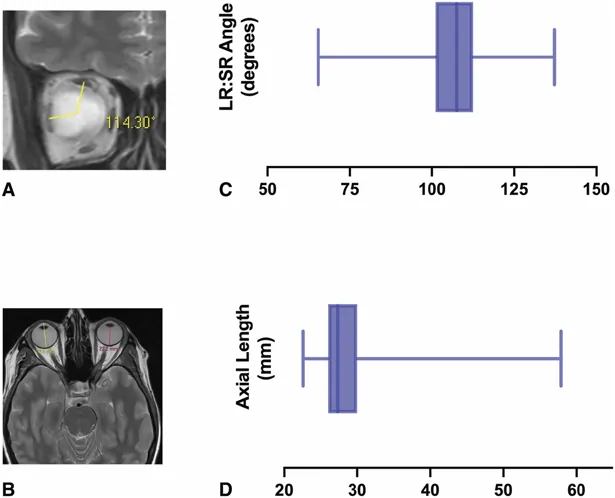
MRI metrics. Illustrative MR scans demonstrating how the SR:LR angle (**A**) and axial length (**B**) were measured. **C**. The LR:SR angle. **D**. Axial length.

A proportion of cases had findings not related to strabismus (or pertinent negatives) on neuroimaging. One case had occipital encephalomalacia secondary to a head injury as a child, another periventricular leukomalacia in association with cerebral palsy, one Arnold Chiari type 1, and another a history of traumatic brain injury. While 2 cases had a history of hyperthyroidism or hypothyroidism (one with a positive thyroglobulin antibodies), neither had clinical or radiographic evidence of thyroid eye disease. No patients in the cohort carried a diagnosis of myasthenia gravis.

### Surgical Intervention

In our cohort, n = 37 patients underwent surgical intervention (Fig. 5), the majority a lateral rectus resection and concurrent medial rectus recession on the same eye with an adjustable suture (n = 20, 54%). A proportion (n = 9, 24%) underwent fixed lateral rectus resection and concurrent medial rectus recession and one patient a Yokoyama procedure.^15^ Three months postsurgery, the majority displayed only latent strabismus or orthophoria (n = 20, 54%), n = 7 had a residual manifest horizontal deviation—all were esotropic (median 6PD for near and 3PD for distance) with only 2 patients having a residual vertical deviation. Nine patients required a second procedure. There was no significant difference between postoperative horizontal measurements at near (Mann–Whitney test, *P* = 0.4257) or distance (Mann–Whitney test, *P* = 0.8177) when comparing fixed suture groups to those having adjustable sutures used.

**FIG. 5.**
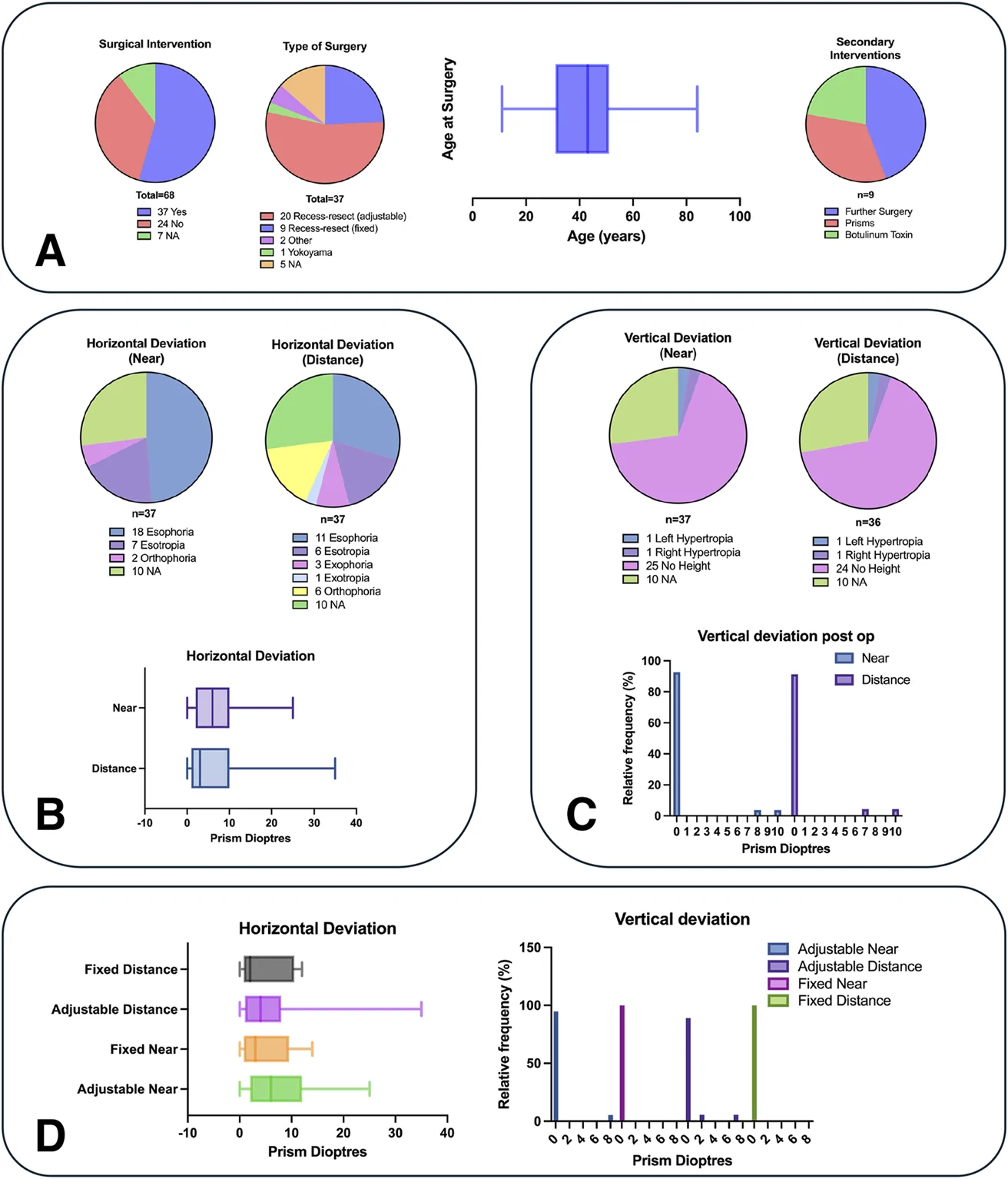
Surgical interventions (**A**) proportion of patients undergoing any surgery, of those, the proportions having each type of procedure. The average age of patients at the time of surgery and the number that required secondary procedures. **B**. Type and magnitude of horizontal deviation at the 3-month postoperative visit. **C**. Type and magnitude of vertical deviation at the 3-month postoperative visit. **D**. Magnitude of horizontal and vertical deviations at 3 months following fixed or adjustable suture approaches to surgery. NA indicates data not available.

One patient in our cohort underwent a Yokoyama procedure;^15^ in fact, this patient had originally presented with a large esotropia and vertical deviation and had undergone a Yokoyama procedure around 2001. They then represented with a recurrent large (45PD) esodeviation around 2010—this was directly following a pars plan vitrectomy for retinal detachment and cataract surgery, which appear to have precipitated the “undoing” of the original Yokoyama suture indicated by marked regression of the superior and lateral muscle bellies from each other. He subsequently underwent a redo Yokoyama, combined with a medial recess recession to good effect (with only 6PD residual esotropia at near and orthophoric in the distance). This residual esotropia did however enlarge over time: the patient declined further surgery but has been well managed for many years with regular medial rectus botulinum toxin injections.

## DISCUSSION

This large clinically and radiographically characterized cohort of patients with esotropia in the context of myopia presents essential real-world data and gives fresh insight into the presentation and management of such patients. It should be borne in mind that this is not a comparative study and that no “nonmyopic” group is described—caution should therefore be exercised in interpretation of causal relationships. That said, this study presents a wealth of useful, real-world data. The cohort reflects the ethnic and gender makeup of the area served in inner London so may be applicable to areas with similar populations. Of note, our cohort was, on average, younger than other published cohorts;^12,17,18,19^ while we did not select for age (other than patients being adults), this may reflect the inclusion of patients with smaller angles of deviation or axial lengths than series restricted to particular esotropic syndromes such as HES^9^ or SES,^13^ which may worsen with age.

Similarly, more permissive inclusion criteria appear to have resulted in a more heterogenous cohort in terms of size of esodeviation—with mean values here smaller than those seen in cohorts restricted to myopic–esotropic syndromes. Similarly, a majority of patients had no significant vertical deviation, or only a very small one, in association with their esodeviation. This also gives a reflection of the relative rarity of presentations of syndromes such as HES.

A lack of an association between magnitude of esodeviation and degree of myopia is superficially surprising—as it may have been supposed that more myopic individuals may manifest larger deviations, especially given the inclusion of those with moderate myopia (3–6D) and those with deviations above 6D (even if the cohort was skewed toward higher myopia, with a median refraction of −8.25D). Indeed, the average radiographic axial length was within the range seen in esomyopic syndromes (median 27.3 mm); however, only 2 cases had a radiographic axial length above the “critical level” (32 mm) described by Aoki et al^12^ where changes in SR:LR anatomy begin to come into play and manifest as HES, which may explain the paucity of HES cases, or those with very large esodeviations. An alternative confounder in this study may come as MRI scans were not specifically performed to quantify axial less and so may have underestimated axial length.

Imaging of SR:LR band anatomy has been proposed to be central in the diagnosis of HES^17^ and its subsequent treatment.^18^ The angle between the 2 muscles on quasi-coronal MR images can serve as a surrogate marker of SR:LR band laxity—with larger angles (around 120°) being more suggestive of HES. In our cohort, the median angle was 107.5°; interestingly, this is somewhat larger than reported values for nonmyopes (97°) and more akin to those with sagging eye syndrome (c. 104°)^17^ or esotropia with staphylomatous myopia (109°)^20^, but still below that of HES (c. 121°).^17,15,21^ It should be remembered that these clinical MRI protocols, unlike research scans, were not specifically designed to produce this measurement (e.g., patients are not given a fixation target during clinical neuro-imaging), which may contribute to this variability. However, these numbers more closely reflect values that will likely be obtained in the clinical setting, without access to research imaging protocols. Nevertheless, this finding was particularly interesting given the lack of patients with HES (n = 2) or documented staphylomas in our cohort, which could have skewed this deviation—perhaps suggesting that SR:LR anatomy is important beyond esomyopic syndromes such as HES.

While the single patient in our cohort undergoing Yokoyama procedure was indeed reported to have SR:LR laxity on imaging, the relative scarcity of patients undergoing the procedure in the cohort was initially surprising. This reinforces previous assertions^17^ that imaging in patients with myopia and esotropia is important to identify SR:LR laxity and ensure that the appropriate surgical procedure is selected.

A further notable surgical feature of this cohort is the relatively large number of patients undergoing fixed or nonadjustable suture surgery for their esotropia. It is reassuring that these patients do not have significantly different outcomes than those undergoing adjustable surgery. Careful preoperative planning with close attention to binocular function is necessary to select appropriate cases for fixed surgery; however, the benefits can be great—including the ability to undertake surgery under local anesthetic.

Therefore, the results from this cohort would suggest that, if those with anatomically distinct syndromes such as HES are identified, a majority of myopic patients with esotropia will respond well to treatment with titrated recess-resect procedures (the standard procedure for esotropia at our institution) even without adjustable sutures in appropriate cases.

Of course, this is within the context of the limitations of the study as a retrospective series. Particularly, the missing data inherent in such a study design—especially access to MR images—in several cases, a report of MR imaging was available, but not the images to allow a quantification of metrics such as SR:LR angle. One of the major drawbacks of this real-world study is the lack of a nonmyopic comparator group, which, together with the limitations of MRI measurements on clinical scans, highlights the pressing need for future prospective randomized, controlled trials to both validate MRI angle measurements in the clinical setting and compare etiologies of esotropia in myopic patients. The authors hope that the described data can form the basis for planning such trials. In addition, it should be noted that the cohort is from a dedicated single specialty eye hospital so may under-represent patients who are systemically unwell or with wider syndromic presentations, which may be seen at multispecialty practices or hospitals. Similarly, as patients often traveled large distances for follow-up, this was generally terminated at 3 months (with care reverting to the referring, local practitioners), thus limiting the longer term conclusion that can be drawn from this study. In addition, the prevailing practice at our institution has been to use recess-resect procedures to operate on a single eye at a time in most cases of esotropia. While excellent surgical outcomes are recorded in our unit, this choice of procedure may provide a barrier in applicability to practices where bimedial recession is the prevailing surgical technique—as is becoming increasingly common worldwide.

In conclusion, despite these limitations, this large, real-world cohort of myopic patients presenting with esotropia gives valuable insight to the clinician. Emphasizing that while it is important to consider special forms of strabismus—such as HES—these are relatively rare compared with more ordinary forms of strabismus, which respond well to traditional surgical intervention.

STATEMENT OF AUTHORSHIP

Conception and design: M. J. Gilhooley, K. Williams, A. Das, I. Davagnanam; Acquisition of data: M. J. Gilhooley, O. Sarwani, G. Johnson, A. Das, K. Williams; Analysis and interpretation of data: M. J. Gilhooley, O. Sarwani, K. Williams. Drafting the manuscript: M. J. Gilhooley; Revising the manuscript for intellectual content: M. J. Gilhooley, O. Sarwani, I. Davagnanam, K. Williams. Final approval of the completed manuscript: All authors.

## Supplementary Material

## References

[1] Bourne RR Stevens GA White RA , Vision Loss Expert Group. Causes of vision loss worldwide, 1990-2010: a systematic analysis. Lancet Glob Health. 2013;1:e339–e349. 10.1016/S2214-109X(13)70113-X 25104599

[2] Williams KM Bertelsen G Cumberland P , European Eye Epidemiology E3 Consortium. Increasing prevalence of myopia in Europe and the impact of education. Ophthalmology. 2015;122:1489–1497. 10.1016/j.ophtha.2015.03.018 PMC4504030 25983215

[3] Dolgin E. The myopia boom. Nature. 2015;519:276–278. 10.1038/519276a 25788077

[4] Holden BA Fricke TR Wilson DA . Global prevalence of myopia and high myopia and temporal trends from 2000 through 2050. Ophthalmology. 2016;123:1036–1042. 10.1016/j.ophtha.2016.01.006 26875007

[5] Wong TY Ferreira A Hughes R Carter G Mitchell P. Epidemiology and disease burden of pathologic myopia and myopic choroidal neovascularization: an evidence-based systematic review. Am J Ophthalmol. 2014;157:9–25.e12. 10.1016/j.ajo.2013.08.010 24099276

[6] Haarman AEG Enthoven CA Tideman JWL Tedja MS Verhoeven VJM Klaver CCW. The complications of myopia: a review and meta-analysis. Invest Ophthalmol Vis Sci. 2020;61:49. 10.1167/iovs.61.4.49 PMC7401976 32347918

[7] Tideman JW Snabel MC Tedja MS . Association of axial length with risk of uncorrectable visual impairment for Europeans with myopia. JAMA Ophthalmol. 2016;134:1355–1363. 10.1001/jamaophthalmol.2016.4009 27768171

[8] Tanaka A Ohno-Matsui K Shimada N . Prevalence of strabismus in patients with pathologic myopia. J Med Dental Sci. 2010;57:75–82. 20437768

[9] Ward DM. The heavy eye phenomenon. Trans Ophthalmol Soc U K (1962). 1967;87:717–726. 5255254

[10] Ohta M Iwashige H Hayashi T Maruo T. Computed tomography findings in convergent strabismus fixus. Nippon Ganka Gakkai Zasshi. 1995;99:980–985. 7676901

[11] Krzizok TH Schroeder BU. Measurement of recti eye muscle paths by magnetic resonance imaging in highly myopic and normal subjects. Invest Ophthalmol Vis Sci. 1999;40:2554–2560. 10509649

[12] Aoki Y Nishida Y Hayashi O . Magnetic resonance imaging measurements of extraocular muscle path shift and posterior eyeball prolapse from the muscle cone in acquired esotropia with high myopia. Am J Ophthalmol. 2003;136:482–489. 10.1016/s0002-9394(03)00276-9 12967802

[13] Rutar T Demer JL. “Heavy eye” syndrome in the absence of high myopia: a connective tissue degeneration in elderly strabismic patients. J AAPOS. 2009;13:36–44. 10.1016/j.jaapos.2008.07.008 PMC2728014 18930668

[14] Tan RJ Demer JL. Heavy eye syndrome versus sagging eye syndrome in high myopia. J AAPOS. 2015;19:500–506. 10.1016/j.jaapos.2015.08.012 PMC4688571 26691027

[15] Yamada M Taniguchi S Muroi T Satofuka S Nishina S. Rectus eye muscle paths after surgical correction of convergent strabismus fixus. Am J Ophthalmol. 2002;134:630–632. 10.1016/s0002-9394(02)01587-8 12383836

[16] Yokoyama T Tabuchi H Ataka S Shiraki K Miki T, and Mochizuki K. Transactions: 26th Meeting, European Strabismological Association, Barcelona, Spain, 2000. (2001): 218–22.

[17] Clark RA Miller JM Demer JL. Three-dimensional location of human rectus pulleys by path inflections in secondary gaze positions. Invest Ophthalmol Vis Sci. 2000;41:3787–3797. 11053278

[18] Wabbels B Fricke J Schittkowski M . Yokoyama procedure for esotropia associated with high myopia: real-world data from a large-scale multicentre analysis. Acta Ophthalmol. 2021;99:e1340–e1347. 10.1111/aos.14808 33655633

[19] Chaudhuri Z Demer JL. Long-term surgical outcomes in the sagging eye syndrome. Strabismus. 2018;26:6–10. 10.1080/09273972.2017.1421676 PMC5823780 29297726

[20] Li Y Wei Q Le A Gawargious BA Demer JL. Rectus extraocular muscle paths and staphylomata in high myopia. Am J Ophthalmol. 2019;201:37–45. 10.1016/j.ajo.2019.01.029 PMC6511469 30731081

[21] Shimizu T Hikoya A Komori M Suzuki H Hotta Y Sato M. Recovery of stereoacuity after Yokoyama procedure in patients with highly myopic strabismus with good vision. Am J Ophthalmol Case Rep. 2023;32:101892. 10.1016/j.ajoc.2023.101892 PMC10362249 37484135

